# Synchronization of Sensory Gamma Oscillations Promotes Multisensory Communication

**DOI:** 10.1523/ENEURO.0101-19.2019

**Published:** 2019-10-23

**Authors:** Jonas Misselhorn, Bettina C. Schwab, Till R. Schneider, Andreas K. Engel

**Affiliations:** Department of Neurophysiology and Pathophysiology, University Medical Center Hamburg-Eppendorf, 20246 Hamburg, Germany

**Keywords:** coherence, communication, EEG, gamma, networks, tACS

## Abstract

Rhythmic neuronal activity in the gamma range is a signature of cortical processing and its synchronization across distant sites has been proposed as a fundamental mechanism of network interactions. While this has been shown within sensory streams, we tested whether cross talk between the senses relies on similar mechanisms. Direct sensory interactions in humans (male and female) were studied with a visual–tactile amplitude matching paradigm. In this task, congruent stimuli are associated with behavioral benefits, which are proposed to be mediated by increased binding between sensory cortices through coherent gamma oscillations. We tested this hypothesis by applying 4-in-1 multi-electrode transcranial alternating current stimulation (tACS) with 40 Hz over visual and somatosensory cortices. In phase stimulation (0°) was expected to strengthen binding and thereby enhance the congruence effect, while anti-phase (180°) stimulation was expected to have opposite effects. Gamma tACS was controlled by alpha (10 Hz) and sham stimulation, as well as by applying tACS unilaterally while visual–tactile stimuli were presented lateralized. Contrary to our expectations, gamma tACS over the relevant hemisphere delayed responses to congruent trials. Additionally, reanalysis of EEG data revealed decoupling of sensory gamma oscillations during congruent trials. We propose that gamma tACS prevented sensory decoupling and thereby limited the congruence effect. Together, our results favor the perspective that processing multisensory congruence involves corticocortical communication rather than feature binding. Furthermore, we found control stimulation over the irrelevant hemisphere to speed responses under alpha stimulation and to delay responses under gamma stimulation, consistent with the idea that contralateral alpha/gamma dynamics regulate cortical excitability.

## Significance Statement

Cortical gamma oscillations structure segregated neural activity and were suggested to represent a fundamental mechanism of network communication. While there is ample evidence for the role of long-range gamma synchronization in unisensory processing, its significance in multisensory networks is still unclear. We show that coordinated sensory gamma oscillations play an important role for direct cross-modal interactions and propose that phase synchronization promotes communication between sensory cortices. To that end, we conducted a state-of-the-art multi-electrode transcranial alternating current stimulation experiment designed to modulate coherence between sensory cortices and analyzed connectivity in a previously recorded high-density EEG dataset. By complementing an interventional with an observational method, we provide novel evidence for the role of synchronized gamma oscillations in multisensory communication.

## Introduction

Perceiving the world through distinct sensory channels provides complementary as well as redundant and conflicting information about the environment. To structure these sensory signals, fundamental neuronal computations are concerned with cross-modal matching of sensory signals. On the neuronal and behavioral levels, processing cross-modally congruent stimuli is associated with enhanced efficiency when compared with incongruent or unimodal processing and often coincides with enhanced cortical activity (Ghazanfar and Schroeder, 2006) and behavioral benefits (Spence, 2011). Within sensory systems, such integrative processes likely involve corticocortical synchronization of high-frequency oscillatory activity (Engel et al., 2001; Fries, 2009). For instance, perceptual grouping and feature binding across cortical columns and hemispheric homologs of visual cortex have been shown to involve phase coupling of neuronal gamma band oscillations (Gray et al., 1989; Engel et al., 1991). Relatedly, it was suggested that synchronized oscillations might provide a solution to the binding problem (Tallon-Baudry et al., 1996; Treisman, 1996). Moreover, gamma oscillations have been proposed to constitute a framework that allows transmitting of coherent patterns of neural activity along sensory streams (Fries, 2015). Together, the coordination of gamma oscillations may enable structuring as well as transmitting sensory information within sensory networks and thereby likely plays an important role in orchestrating multisensory interactions (Keil and Senkowski, 2018).

A number of studies have investigated gamma band activity during multisensory perception. Visual stimulus detection, for instance, was shown to be improved by redundant auditory stimuli while gamma band responses in frontal cortex were enhanced (Senkowski et al., 2005, 2007). Recognition and classification of visual objects was improved by congruent auditory input showing increased gamma band power in temporal or parietal cortices (Yuval-Greenberg and Deouell, 2007; Schneider et al., 2008a). While the aforementioned studies showed multisensory modulations of gamma band power in association cortices, other studies also noted changes in sensory cortices (Krebber et al., 2015; Friese et al., 2016). For instance, attention for suprathreshold audio–visual stimuli was associated with enhanced sensory gamma oscillations in both the visual and auditory cortices (Friese et al., 2016) and matching congruent visual–tactile motion stimuli induced enhanced gamma power in visual and somatosensory cortices (Krebber et al., 2015). Additionally, there is evidence for altered gamma oscillations underlying schizophrenia (Uhlhaas and Singer, 2010; Mulert et al., 2011; Curic et al., 2019). In these patients, aberrant multisensory integration was shown to be accompanied by altered gamma band dynamics in response to multisensory stimuli (Stone et al., 2014; Balz et al., 2016). Together, cross-modally corresponding or congruent stimuli typically induce strong local synchronization of gamma band oscillations in both sensory and association cortices.

In addition to local changes in gamma band activity, it was suggested that cross-modal interactions involve interareal phase synchronization of sensory gamma oscillations (Senkowski et al., 2008; Keil and Senkowski, 2018). Specifically, enhanced processing of cross-modally congruent stimuli might imply feature binding across modalities mediated by synchronization of sensory gamma oscillations. A constraint in testing this hypothesis is that differences in power constitute a bias for the computation of phase coherence (Bastos and Schoffelen, 2016). As reviewed above, many multisensory paradigms would, thus, not be suited for testing this prediction. Here, we used a paradigm that has not revealed differences in gamma power in sensory cortices during cross-modal matching (Misselhorn et al., 2019). In this task, participants match concurrent amplitude changes of visual and tactile stimuli that are either congruent (both increase or decrease in intensity) or incongruent (increase in one and decrease in the other modality). Following Senkowski et al. (2008), we assumed that congruence enhancement would entail increased coupling between sensory gamma oscillations. Modulating coupling between visual and somatosensory cortices should therefore influence the effect of cross-modal congruence. To test this hypothesis, we used focal multi-electrode transcranial alternating current stimulation (tACS) to modulate the synchrony of sensory gamma oscillations between visual and somatosensory cortices. Sham-controlled tACS was applied at either 10 Hz (alpha) or 40 Hz (gamma) with 0° (in-phase) or 180° (anti-phase) phase shift between montages. Additionally, we controlled the effect of stimulation by presenting lateralized stimuli and applying tACS unilaterally. We hypothesized task-specific effects of tACS only to occur when electrical and sensory stimulation were targeted at the same hemisphere, but no or only unspecific effects when electrical and sensory stimulation were targeted at different hemispheres. Specifically, we expected in-phase gamma stimulation over the relevant hemisphere to enhance the congruence effect by (1) speeding responses to congruent stimuli due to enhanced feature binding and (2) delaying responses to incongruent stimuli by imposing “false” feature binding. Gamma anti-phase tACS over the relevant hemisphere should show inverse effects. Additionally, we reanalyzed the aforementioned EEG data (Misselhorn et al., 2019) with respect to the coherence of gamma oscillations to inform the interpretation of the behavioral results from this study.

## Materials and Methods

### tACS experiment

#### Participants

Twenty-four participants were recruited and completed a training session, after which four participants dropped out due to insufficient performance (<60% accuracy). Twenty participants completed three experimental sessions (13 females; age, 25.3 ± 4.5 years). None of them had a history of neurologic or psychiatric disorders. All participants gave informed written consent and received monetary compensation for their participation. The local ethics committee approved the study, which was conducted in accordance with the Declaration of Helsinki.

#### Experimental design

**Figure 1.**
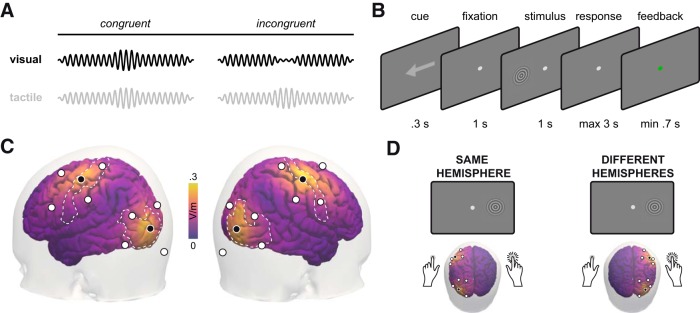
Experimental design. ***A***, visual–tactile stimuli were presented on each trial. Brief changes in stimulus intensity occurred concurrently in both modalities, either in the same direction (“congruent”) or in different directions (“incongruent”). ***B***, Each trial started with a central arrow that cued the left or right side reliably. After 1 s of central fixation, the visual–tactile stimulus was presented on the cued side. Participants were asked to maintain central fixation and report congruence of the presented stimulus. After response, participants received feedback. ***C***, Multi-electrode tACS montage (black and white electrodes represent different polarities) and estimation of current density on cortical surface. Participants received either left or right hemispheric stimulation with two 4-in-1 montages over visual and somatosensory cortices. Color coding on cortical surface corresponds to the simulated maximum absolute field strength in V/m**. *D***, On a given trial, electrical and sensory stimulation could be targeted at the same hemisphere (left) or at different hemispheres (right).

Participants performed a spatially cued cross-modal amplitude matching task on visual–tactile stimuli. We presented a circular, expanding grating (diameter, 5° visual angle) on a CRT screen (refresh rate, 120 Hz; model HM204DTA, Iiyama) against a gray background as visual stimulation. Gratings were presented with 5° visual angle offset to the left or right of the vertical meridian. Tactile stimulation was realized by a high-frequency vibration delivered to the fingertips of both index fingers (250 Hz on C-2 tactors, Engineering Acoustics). Throughout the whole experiment participants kept fixation on a central fixation point. To induce a covert shift of attention, a centrally presented arrow cued the left or right side (100% reliability, 300 ms; Fig. 1*B*). After 1 s, a visual–tactile stimulus was presented on the cued side only. That is, visual stimuli were presented to the left or right side of the fixation, and the tactile stimulus was presented to the left or right index finger. On each trial, both visual and tactile components underwent a brief suprathreshold change in intensity, either an increase or a decrease (Fig. 1*A*). Magnitudes of change were derived from a previous behavioral study using a similar paradigm (Misselhorn et al., 2016). Participants were asked to compare the change direction between visual and tactile components and report whether they changed congruently or incongruently (Fig. 1*A*). Responses were instructed to be given as fast as possible by using a foot switch. After a training session, participants completed two identical experimental sessions containing three blocks holding 192 trials. Experimental session used either alpha (10 Hz) or gamma (40 Hz) stimulation. These canonical stimulation frequencies were chosen because previous studies showed behavioral as well as neurophysiological effects for these frequencies (Helfrich et al., 2014; Schwab et al., 2019). The order of experimental sessions was counterbalanced across participants. Experimental blocks featured in-phase, anti-phase, or sham stimulation (for details, see Electrical stimulation). The order of stimulation conditions was counterbalanced across participants.

#### Electrical stimulation

Alternating currents were administered in 4-in-1 montages with current flow between the four outer electrodes and one central electrode (Patel et al., 2009; Saturnino et al., 2015) using Ag/AgCl ring electrodes (diameter, 12 mm). This configuration results in focal electric fields with peaks underneath the central electrode (Fig. 1*C*). For each participant, we prepared two of these montages designed to target primary visual and primary somatosensory cortices of one hemisphere, respectively. The side of stimulation was counterbalanced across participants. In conjunction with the lateralized experimental design, this resulted in equal proportions of trials in which electrical and sensory stimulation were targeted at the same hemisphere (Fig. 1*D*, left) or at different hemispheres (Fig. 1*D*, right). Before experimental blocks, stimulation was ramped up to 2 mA peak to peak within 10 s. Sham blocks started with the same ramps but included no stimulation thereafter. For in-phase stimulation, we used the same waveforms for both montages. For anti-phase stimulation, one waveform was shifted by 180°. Two separate DC stimulators were used (DC-Stimulator Plus, Neuroconn). Stimulators were operated in external mode, allowing control of the current output via voltage input. The voltage signal was computed in Matlab and produced by a NI-DAQ device run with Labview (NI USB 6343, National Instruments). Impedances of each of the four outer electrodes relative to the central electrode were kept comparable within montages (10–100 kΩ). This is crucial because identical impedances were assumed for the simulation of electric fields.

#### Simulation of electric fields

Electrode positions for the 4-in-1 montages were chosen such that electric field strength was maximized in visual and somatosensory areas. Simulations of current flow were performed based on the lead field matrix L, which was computed for a realistic three-shell head model (Nolte and Dassios, 2005), and a cortical grid in MNI space, obtained by downsampling the Freesurfer template to 10,000 grid points (Desikan et al., 2006). The electric field at location *x* was estimated by linear weighting of the lead field matrix L with the injected currents $\alpha_{i}$, where *i* denotes indices of the 10 stimulation electrodes, as follows:

$$\vec{E}\left(\vec{x}\right)=\underset{i}{\sum }(\vec{L}(\vec{x})\alpha_{i}).$$

Within visual and somatosensory regions, peak values of 0.3 V/m were reached using currents with peak values of 1 mA (2 mA peak to peak). Focality was high as field strengths rapidly decreased when moving away from the central electrode (Fig. 1*C*). This ensured that effective electrical stimulation was confined to the targeted regions of one hemisphere only.

#### Statistical analysis

The effects of tACS were evaluated by analyzing accuracy and response times (RTs). First, we computed a repeated measures ANOVA with factors *HEMISPHERE* (same/different), *FREQUENCY* (alpha/gamma), *STIMULATION* (sham/in-phase/anti-phase), and *CONGRUENCE* (congruent/incongruent). Where necessary, the Greenhouse–Geisser correction was applied. Tables containing complete results from ANOVA are provided in Extended Data Table 1-1. Higher-order interactions were followed up by computing reduced ANOVA models and, finally, significant two-way interactions were followed up by a nonparametric analysis based on comparing RT distributions. To that end, we estimated cumulative distribution functions (CDFs) of RT distributions using a Gaussian kernel estimator (Botev et al., 2010). CDFs were estimated for RTs between 0 and 4 s using 1024 bins for each subcondition and participant. Next, we computed differences between CDFs and averaged across participants. To decide about the statistical significance of differences between CDFs, we constructed confidence intervals (CIs) by permutation tests. That is, we shuffled all data from a given interaction into two sets, computed CDFs and stored the difference between the CDFs of the two sets as the null-distribution (100,000 permutations). Two-sided CIs were constructed by finding percentiles (lower bound, α/2; upper bound, 100-α/2) in the null distribution that reflect the range of positive and negative differences along the RT range that can be expected to result from random fluctuations. The final CIs are corrected for both (1) multiple testing due to condition contrasts and (2) multiple tests along the RT range, with an initial probability of false positives set to α = 5%. The latter source of multiple tests is especially critical because testing a range of values compared with testing one value (e.g., a central tendency as in ANOVA) profoundly inflates the probability of false positives. The first issue was dealt with by Bonferroni correction and yields α. The second issue, however, would not be adequately dealt with by Bonferroni correction because the number of tests along the RT range is an arbitrary choice. Thus, instead of applying the α at each RT bin separately, we applied it globally to all RT bins collectively. That is, we counted instances of the null distribution (one instance is the null result from a single permutation) that fall outside the confidence interval at any RT bin. The global α was found by iteratively decreasing α until only the α percentage of all null distribution instances fell outside the confidence interval. Thus, even small deflections outside the confidence interval represent statistically robust effects. The resulting α levels will be reported.

#### Analysis of tACS side effects

After each experimental block featuring a given stimulation condition, participants completed a questionnaire designed to reflect (1) the perceived maximum intensity of skin sensations (itching, warmth, stinging, pulsating), phosphenes, fatigue, and pain (ranked as either “absent”/0, “light”/1, “moderate”/2, “pronounced”/3, or “strong”/4) as well as (2) the timecourse of sensations (“beginning,” “end,” “always”). Condition differences in perceived intensity were evaluated using Wilcoxon matched-pairs signed-rank tests without applying a correction for multiple comparisons to maximize power for detecting possibly biasing differences between conditions. Skin sensations were aggregated by computing median responses over the four qualities. To analyze whether participants were blinded or whether they could perceive the difference between sham and verum, we computed a binary score reflecting whether participants perceived peripheral sensations only in the beginning (0) or all the time (1). We report averages that can be interpreted as fractions and uncorrected *p* values from McNemar’s tests. Finally, we assessed whether significant tACS-related behavioral effects detected in the main analysis could be explained by the perceived intensity of sensations. To that end, we ranked individual behavioral effects and correlated these scores with the questionnaire data by means of Spearman correlations.

### Analysis of EEG data

An exhaustive description of experimental procedure and data can be found in Misselhorn et al. (2019).

#### Participants

Twenty-one participants (11 females; age, 23.8 ± 2.5 years) were invited for two sessions of EEG. None of them had a history of neurologic or psychiatric disorders. All participants gave informed written consent and received monetary compensation for their participation. The local ethics committee approved the study, which was conducted in accordance with the Declaration of Helsinki.

#### Experimental design

Participants received trimodal sensory stimulation (for details, see Stimulus material) on each trial of the experiment. These trimodal stimuli contained a visual, an auditory, and a tactile component. On each trial, all components underwent a brief intensity change. That is, visual contrast, auditory loudness, and vibration strength were either increased or decreased. The task was to attend bimodal pairs [visual–tactile (VT) or audio–visual (AV)] blockwise and compare attended intensity changes. These changes could be either congruent (i.e., in the same direction) or incongruent (i.e., in different directions); the respective third modality had to be ignored. Participants responded verbally after stimulus offset. Blocks of VT and AV attention contained 64 trials with equal contributions of the eight possible stimulus configurations of increases and decreases across modalities. On 2 separate days, 10 blocks each of VT and AV attention were performed in an alternating fashion, summing up to 1280 trials.

#### Stimulus material

Visual stimulation consisted of a circular, expanding grating presented centrally on a CRT screen (refresh rate, 120 Hz; model HM204DTA, Iiyama) with gray background spanning a visual angle of 5°. The auditory stimulus component was a complex sinusoidal tone (13 sine waves: 64 Hz and its first 6 harmonics as well as 91 Hz and its first five harmonics; low-frequency modulator, 0.8 Hz) played back with audiometric insert earphones binaurally at 70 dB (E-A-RTONE 3A, 3M). The tactile component was a high-frequency vibration delivered to the fingertips of both index fingers (250 Hz on C2 tactors, Engineering Acoustics). Visual contrast, auditory loudness, and vibration amplitude were experimentally modulated. In total, trimodal stimuli had a fixed duration of 2 s, and changes in intensity lasted for 300 ms. Transitions were smoothed with cosine tapers, and onsets were jittered across trials between 700 and 1000 ms after stimulus onset. The magnitude of change per modality and change direction was estimated individually with a psychometric step function before experimental blocks on each day (Watson and Pelli, 1983).

#### Processing of EEG data

High-density EEG was recorded from 128 channels using active Ag/AgCl electrodes referenced to the nose (EasyCap) via BRAINAMP MR amplifiers (Brain Products) and digitized after analog filtering by the amplifier (hardware settings: low cutoff, 10 s (time constant); high cutoff, 450 Hz; sampling rate, 1000 Hz). After resampling to 500 Hz, data were filtered using the default settings of the EEGLAB function pop_eegfiltnew.m, which uses Hamming window sinc FIR (finite impulse response) filters and estimates the filter order [bandpass, 30–120 Hz (order 220); notch, 49–51 Hz, 99–101 Hz (order 1650); Delorme and Makeig, 2004]. Due to low signal-to-noise ratio, 19 electrodes of the outer rim covering neck and chin were excluded from further analysis. Before preprocessing, data were rereferenced to the common average and cut into epochs locked to stimulus onset (−500 to 2000 ms). Due to 1/f properties of cortical activity, an EEG signal >30 Hz is usually dominated by muscle activity. Thus, we used independent component (IC) analysis to identify components of cortical gamma activity by evaluating topography, time course, and spectrum of all ICs (Hipp and Siegel, 2013). Additionally, we identified ICs related to muscle activity underlying miniature saccades (Hassler et al., 2011). We rejected all components that did not show clear characteristics of cortical gamma activity as well as the ICs reflecting miniature saccades (rejected ICs, 66 ± 11%). Stratified data held, on average, 426 ± 89 epochs per participant. We divided data for each participant into two sets based on the congruence of changes in the visual and tactile stimuli. That is, irrespective of attention condition, congruent trials featured trimodal stimuli in which the visual and tactile components changed in the same direction. Conversely, incongruent trials contained all trials with stimuli in which the visual and tactile components changed in different directions. In sensor space, event-related potentials were averaged per experimental condition and subtracted from single-trial data. Source reconstruction was performed with exact low-resolution electromagnetic tomography (eLORETA; regularization, 0.05; Pascual-Marqui et al., 2011). Spatial filters were constructed using a three-shell head model (Nolte and Dassios, 2005) and a cortical grid in MNI space obtained by downsampling the Freesurfer template to 10,000 grid points (Desikan et al., 2006). Dipole directions were chosen by finding the direction of maximum power at 40 Hz, with singular value decomposition for each node in the cortical surface. Power was estimated by computing auto-spectra using fast Fourier transform (fft.m function in Matlab) based on all trials of a given condition and participant.

#### Statistical analysis

To compute the time courses of power and the coherence of sensory gamma oscillations, we used a sliding window approach with Hanning windows of length 1 s, which were shifted in 50 ms steps from −1000 to 1000 ms relative to change onset. In the resulting 21 windows, we computed cross-spectra for the whole cortical grid from each trial and subsequently averaged them. From the trial-averaged cross-spectra, we computed imaginary coherence (iCoh; Nolte et al., 2004) among all cortical nodes as well as power at each node at 40 Hz (1 Hz resolution). Finally, power was averaged across all nodes of the left and right visual and somatosensory cortices based on the anatomic atlas of Freesurfer (Desikan et al., 2006). iCoh was averaged for all intrahemispheric connections between visual and somatosensory cortices ignoring all intraregional as well as interhemispheric connections. Each individual time course of power or iCoh was normalized by *z*-transform based on all data points of a given time course. Additionally, we subtracted a baseline from −500 to −250 ms relative to change onset (note: time points are referred to by their center bin). Statistical comparison with baseline and between conditions was performed by averaging time course data for the time points of change (0–300 ms) and computing one-sample *t* tests within conditions and paired-sample *t* tests across conditions. No correction for multiple tests was applied to have maximal power for detecting possibly biasing differences in power. iCoh was analyzed similarly by averaging for the epoch of change (0–300 ms) and performing *t* tests as described before. Here, we corrected for multiple testing according to Bonferroni.

## Results

### ANOVA on behavioral outcomes during tACS

**Figure 2.**
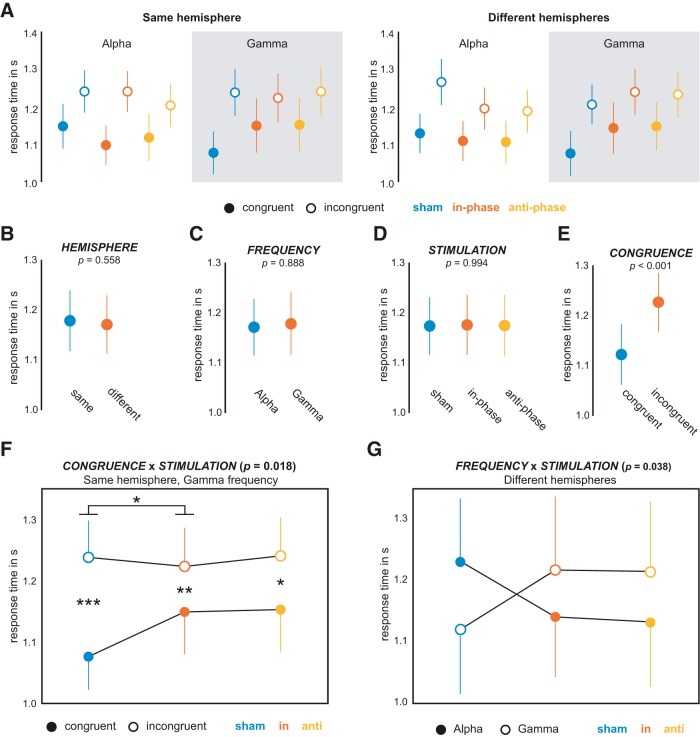
Results from ANOVA. A correlation analysis between tACS side effect and significant interactions in the RT ANOVA is provided as Extended Data Figure 2-1. ***A***, Overview of all conditions entering the *HEMISPHERE* (2) × *FREQUENCY* (2) × *STIMULATION* (3) × *CONGRUENCE* (2); repeated-measures ANOVA (rmANOVA). Filled/empty circles represent congruent/incongruent trials. Factor levels of *STIMULATION* are color coded (blue, sham; red, in-phase; yellow, anti-phase). ***B–E***, Main effects resulting from 2 × 2 × 3 × 2 rm-ANOVA. ***F***, *CONGRUENCE* × *STIMULATION* interaction for gamma stimulation over the hemisphere targeted by sensory stimuli (*p < 0.05, **p < 0.01, ***p < 0.001). ***G***, *FREQUENCY* × *STIMULATION* interaction for stimulation of the hemisphere not targeted by sensory stimulation.

A complete repeated measures ANOVA model with factors *HEMISPHERE* (same/different), *FREQUENCY* (alpha/gamma), *STIMULATION* (sham/in-phase/anti-phase), and *CONGRUENCE* (congruent/incongruent) was computed for both accuracy and RTs. Participants were well trained on the task and gave, on average, correct responses in ∼83% of all trials. Accuracy differed significantly between congruent and incongruent trials (*F*_(1,19)_ = 10.122, *p* = 0.005, $\eta_{p}^{2}$ = 0.348), and errors were less likely in congruent trials (85.48%) when compared with incongruent trials (80.36%). No other factor or any interaction significantly influenced accuracy (Extended Data Table 1-1). The timing of responses broken down by condition is shown in Figure 2*A*. RTs showed a similar, but stronger effect of *CONGRUENCE* (Fig. 2*E*; *F*_(1,19)_ = 34.659, *p* = 1.142 * 10^−5^, $\eta_{p}^{2}$ = 0.646). That is, responses in congruent trials were on average faster than in incongruent trials (mean RT difference, 105 ms). In contrast to accuracy, all other factors significantly affected RTs, resulting in significant two-, three-, and four-way interactions (Table 1, Extended Data Table 1-1). To resolve these high-order interactions, we computed reduced ANOVA models until interpretable two-way interactions remained.

**Table 1:** Significant effects of all computed ANOVA models on response times

Factor	*F*	*p*	$\eta_{p}^{2}$
Complete ANOVA model			
CONGRUENCE	34.659	0.000	0.646
STIMULATION × CONGRUENCE	4.199	0.032	0.181
FREQUENCY × STIMULATION × CONGRUENCE	4.089	0.027	0.177
HEMISPHERE × FREQUENCY × STIMULATION × CONGRUENCE	4.862	0.015	0.204
Reduced model: different HEMISPHEREs			
CONGRUENCE	24.803	0.000	0.566
FREQUENCY × STIMULATION	3.771	0.038	0.166
Reduced model: same HEMISPHERE			
CONGRUENCE	40.028	0.000	0.678
FREQUENCY × STIMULATION × CONGRUENCE	7.548	0.002	0.284
Reduced model: same HEMISPHERE, alpha FREQUENCY			
CONGRUENCE	43.454	0.000	0.696
Reduced model: same HEMISPHERE, gamma FREQUENCY			
CONGRUENCE	26.133	0.000	0.579
STIMULATION × CONGRUENCE	4.578	0.018	0.194

Complete tables of ANOVA for accuracy and response time data can be found as Extended Data Table 1-1.

10.1523/ENEURO.0101-19.2019.f2-1 Figure 2-1 Side effects of tACS. ***A***, Visualization of questionnaire data for skin sensations (aggregated across itching, warmth, stinging, pulsating), phosphenes, fatigue, and pain. Lowest row represents “absent” response while top rows indicate “light” to “strong” sensation. The sizes of circles represent the number of responses, and asterisks indicate the median response per condition and sensation. ***B–D***, Correlations of ranked behavioral effect detected in ANOVA with skin sensations (rank 1 is lowest value). Bar plots indicate direction of behavioral effects. Across all effects, correlations are weak and nonsignificant, but show signs that are opposite to what would have been expected if side effects drove the behavioral effects. Download Figure 2-1, EPS file.

10.1523/ENEURO.0101-19.2019.t1-1 Table 1-1 : Complete and reduced ANOVA results from behavior in tACS experiment Download Table 1-1, DOC file.

First, we resolved the factor *HEMISPHERE* by computing separate ANOVAs with factors *FREQUENCY*, *STIMULATION*, and *CONGRUENCE*. For stimulation over the hemisphere not targeted by sensory stimulation (DIFFERENT), we found a significant effect of *CONGRUENCE* (*F*_(1,19)_ = 24.803, *p* = 8.308 * 10^−5^, $\eta_{p}^{2}$ = 0.566) as well as an interaction between *FREQUENCY* and *STIMULATION* (*F*_(2,38)_ = 3.771, *p* = 0.038, $\eta_{p}^{2}$ = 0.166). *Post hoc* comparisons revealed opposing effects for alpha and gamma stimulation, as follows: under alpha stimulation, RTs shortened from sham to both in-phase and anti-phase stimulation. Under gamma stimulation, RTs were prolonged from sham to both in-phase and anti-phase stimulation. None of these differences, however, were significant after correction for multiple comparisons (all *p* > 0.2; Fig. 2*G*).

For stimulation over the hemisphere that was targeted by sensory stimulation (SAME), we found a significant effect of *CONGRUENCE* (*F*_(1,19)_ = 40.028, *p* = 4.518 * 10^−6^, $\eta_{p}^{2}$ = 0.678) as well as an interaction among *FREQUENCY*, *STIMULATION*, and *CONGRUENCE* (*F*_(2,38)_ = 7.548, *p* = 0.002, $\eta_{p}^{2}$ = 0.284). This three-way interaction was investigated by the resolving factor *FREQUENCY*. For alpha frequency, only *CONGRUENCE* significantly affected RTs (*F*_(1,19)_ = 43.454, *p* = 2.614 * 10^−6^, $\eta_{p}^{2}$ = 0.696). In contrast, a reduced ANOVA for gamma frequency showed, next to an effect of *CONGRUENCE* (*F*_(1,19)_ = 26.133, *p* = 6.194 * 10^−5^, $\eta_{p}^{2}$ = 0.579), an interaction between *STIMULATION* and *CONGRUENCE* (*F*_(2,38)_ = 4.578, *p* = 0.018, $\eta_{p}^{2}$ = 0.194). *Post hoc* comparisons showed significant effects of *CONGRUENCE* under all stimulation conditions (Bonferroni-corrected *p* values; sham: *t*_(19)_ = −5.099, *p* = 0.0002; in-phase: *t*_(19)_ = −3.516, *p* = 0.0069; anti-phase: *t*_(19)_ = −3.049, *p* = 0.0198). Pairwise comparisons between stimulation conditions showed that the effect of *CONGRUENC*E was significantly smaller under in-phase compared with sham stimulation (Bonferroni-corrected *p* values; sham vs in-phase: *t*_(19)_ = −2.630, *p* = 0.0495; sham vs anti-phase: *t*_(19)_ = −2.325, *p* = 0.0939; in-phase vs anti-phase: *t*_(19)_ = 0.5338, *p* = 1.7991). Specifically, responses to congruent stimuli were delayed while responses to incongruent stimuli did not show significant differences to sham (Fig. 2*F*).

### Nonparametric follow-up analysis of response times distributions

Significant two-way interactions were followed up by comparisons of CDFs of RT data that were evaluated using nonparametric permutation statistics. This approach represents a powerful investigation of subtle changes in the shape of distributions that do not necessarily result in significant changes of mean values. It should be noted that correction for multiple comparisons was performed for both the number of condition-wise comparisons and the range of RTs, resulting in a conservative alpha value (alpha = 0.000129). Condition differences exceeding the confidence interval, even for narrow RT ranges, thus represent statistically robust effects.

**Figure 3.**
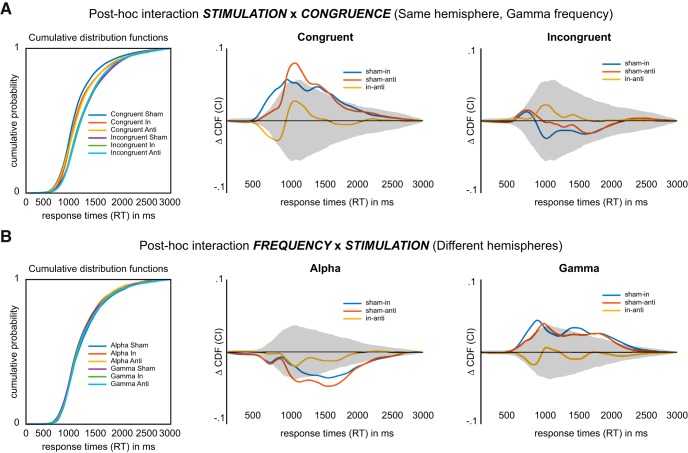
Results from follow-up analysis comparing RT distributions. ***A***, Follow-up analysis of the *STIMULATION* × *CONGRUENCE* interaction found for gamma stimulation over the hemisphere targeted by sensory input (same). Left, CDFs corresponding to all levels of the 2 × 3 interaction. Middle, Differences in CDFs for congruent trials between levels of factor *STIMULATION*. Gray-shaded area indicates the CI as estimated by nonparametric permutation statistics (corrected for multiple comparisons, *p* < 0.000129). All differences outside the CI indicate significant differences between the respective conditions. Right, Differences in CDFs for incongruent trials between levels of factor *STIMULATION*. ***B***, Follow-up analysis of the *FREQUENCY* × *STIMULATION* interaction found for stimulation over the hemisphere not targeted by sensory input. Left, CDFs corresponding to all levels of the 2 × 3 interaction. Middle, Differences in CDFs for trials under alpha stimulation between levels of factor *STIMULATION*. Gray-shaded area indicates the CI as estimated by permutation statistics (corrected for multiple comparisons, *p* < 0.000129). Right, Differences in CDFs for trials under gamma stimulation between levels of factor *STIMULATION*.

The interaction between *STIMULATION* and *CONGRUENCE* for stimulation of the hemisphere targeted by sensory input (SAME) was followed up by subtracting CDFs of stimulation conditions pairwise separately for congruent and incongruent trials. For congruent trials, both in-phase and anti-phase stimulation differed significantly from sham, meaning that responses were slowed down by stimulation (sham vs in-phase: 440–960 and 1330–1660 ms; sham vs anti: 880–1610 ms; Fig. 3*A*, middle). Over and above the effects detectable by ANOVA, we found a difference between in-phase and anti-phase stimulation (430–710 ms; Fig. 3*A*, middle). Accordingly, in-phase stimulation slowed responses more strongly when compared with anti-phase stimulation. The same analysis for incongruent trials did not show any differences with respect to stimulation conditions (Fig. 3*A*, right).

The interaction between *FREQUENCY* and *STIMULATION* for stimulation of the hemisphere not targeted by sensory input (DIFFERENT) was followed up by subtracting CDFs of stimulation conditions pairwise separately for alpha and gamma stimulation. For alpha stimulation, we found significant differences between sham and in-phase stimulation as well as between sham and anti-phase stimulation corresponding to a speeding of responses (sham vs in-phase: 180–670 and 1330–2280 ms; sham vs anti-phase: 450–690 and 1010–2215 ms; Fig. 3*B*, middle). For gamma stimulation, we found significant differences between sham and in-phase as well as sham and anti-phase stimulation corresponding to a slowing of responses (sham vs in-phase: 525–970 and 1330–2240 ms; sham vs anti-phase: 865–1015 and 1615–2040 ms; Fig. 3*B*, right). For both frequencies, we did not find significant differences between in-phase and anti-phase stimulation.

### Side effects of tACS

Most participants reported tACS-related side effects (Extended Data Fig. 2-1). While most participants reported “light” to “strong” skin sensations (median ± interquartile range, 1 ± 1.25) only three participants reported phosphenes (0 ± 0). Fatigue (0 ± 1) and pain (0 ± 1) were absent in the majority of participants. Importantly, the intensity of sensations overall did not differ with respect to sham, in-phase, or anti-phase stimulation (uncorrected, for all, *p* > 0.09) and also showed no differences with respect to stimulation frequency (uncorrected, for all, *p* > 0.38). Next to the intensity of sensations, we asked for the timecourse of a given sensation and coded responses into a binary decision for initial (0) or constant (1) stimulation (averages; 10 Hz: sham = 0.32, in-phase = 0.68, anti-phase = 0.74; 40 Hz: sham = 0.32, in-phase = 0.68, anti-phase = 0.63). Differences in the timecourse of perception indicative of sham and verum conditions were found significant or trending for both alpha and gamma stimulation (all uncorrected; alpha: sham vs in-phase, *p* = 0.03; sham vs anti-phase, *p* = 0.02; in-phase vs anti-phase, *p* = 0.87; gamma: sham vs in-phase, *p* = 0.03; sham vs anti-phase, *p* = 0.07; in vs anti-phase, *p* = 0.87). Sorensen–Dice similarity coefficients showed that ratings for alpha and gamma stimulation were comparable (sham, 1; in-phase, 0.77; anti-phase, 0.77). Finally, correlations among the average effect values of all three significant interactions detected with ANOVA and the overall intensity of skin sensations were weak and nonsignificant (all |*r*| < 0.25 and all *p* > 0.3). Interestingly, extreme values for skin sensations were more likely to occur in participants with weak behavioral effects. Nonsignificant correlations thus showed opposite signs than would have been expected if the strength of sensations was indicative of behavioral effect size.

### EEG data

**Figure 4.**
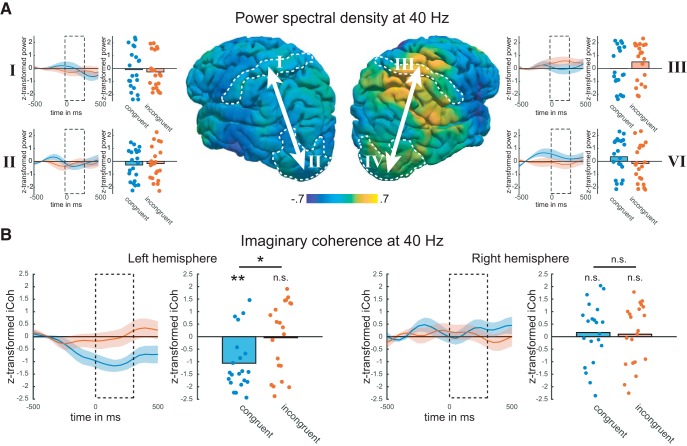
Results from reanalysis of EEG data. In a previous study (Misselhorn et al., 2019), EEG was recorded during a similar experimental paradigm. Here, we reanalyzed the data with a focus on power and imaginary coherence in and between visual and somatosensory areas at 40 Hz. In the extended data, we present an analysis comparing the average change in 40 Hz power across hemispheres (Extended Data Fig. 4-1) as well as an analysis of the correlation between power and iCoh (Extended Data Fig. 4-2). *A*, Middle, Distribution of average normalized power at 40 Hz during the change interval. Dotted outlines indicate borders of ROIs. Left, right, Roman numerals indicate the ROI in which power was computed. Each graph depicts the time course of power changes for congruent (blue) and incongruent (red) trials as well as the average power during change (dotted box) as a bar/scatter plot. All comparisons resulted in nonsignificant differences (all *p* > 0.05). ***B***, Left, Timecourse of iCoh between visual (II) and somatosensory (I) cortices of the left hemisphere. Bar/scatter plots depict average iCoh during change interval (dotted box; n.s. *p* > 0.05, *p < 0.05, **p < 0.01). Right, Timecourse of iCoh between visual (IV) and somatosensory (III) cortices of the right hemisphere. Bar/scatter plots depict average iCoh during change interval.

A connectivity analysis of EEG data previously recorded during a similar task was used to guide interpretation of the tACS effects on behavior (Misselhorn et al., 2019). Time series of cleaned EEG data were projected to source space to analyze local and interareal synchronization of gamma oscillations at 40 Hz in and between early visual and somatosensory regions. First, we analyzed power to identify potential biases to the evaluation of iCoh (Nolte et al., 2004). All statistics of control analyses are reported without correction for multiple comparisons to have maximal power for the detection of potentially small, but biasing differences in power. During the change interval, condition-averaged 40 Hz gamma power was relatively increased in the right hemisphere and, to a smaller degree, suppressed or unchanged in the left hemisphere (Fig. 4*A*, middle). Statistical analysis of power change in the visual and somatosensory regions of interest (ROIs) showed that these trends were not significant (Extended Data Fig. 4-1; left visual: *t*_(20)_ = −1.506, *p* = 0.148; left somatosensory: *t*_(20)_ = −0.891, *p* = 0.384; right visual: *t*_(20)_ = 0.561, *p* = 0.581; right somatosensory: *t*_(20)_ = 1.147, *p* = 0.265). Furthermore, power changes did not differ significantly between hemispheres (visual left-right: *t*_(20)_ = −1.292, *p* = 0.211; somatosensory left-right: *t*_(20)_ = −1.724, *p* = 0.100). Also, condition-averaged 40 Hz power did not correlate with condition-averaged iCoh for left and right visual ROIs as well as left somatosensory ROI (Extended Data Fig. 4-2; left visual: *r*_(19)_ = −0.368, *p* = 0.111; right visual: *r*_(19)_ = −0.145, *p* = 0.532; left somatosensory: *r*_(19)_ = −0.199, *p* = 0.388), but showed a negative correlation for right somatosensory ROI (Extended Data Fig. 4-2; right somatosensory: *r*_(19)_ = −0.532, *p* = 0.013). Importantly, statistical comparison between congruent and incongruent trials did also not show any significant differences (Fig. 4*A*: I, left somatosensory: congruent, *t*_(20)_ = −0.241, *p* = 0.812; incongruent, *t*_(20)_ = −0.848, *p* = 0.407; congruent-incongruent, *t*_(20)_ = 0.396, *p* = 0.697; II, left visual: congruent, *t*_(20)_ = −1.084, *p* = 0.291; incongruent, *t*_(20)_ = −0.613, *p* = 0.547; congruent-incongruent, *t*_(20)_ = −0.259, *p* = 0.798; III, right somatosensory: congruent, *t*_(20)_ = 0.001, *p* = 0.999; incongruent, *t*_(20)_ = 1.592, *p* = 0.127; congruent-incongruent, *t*_(20)_ = −1.070, *p* = 0.298; IV, right visual: congruent, *t*_(20)_ = 1.151, *p* = 0.263; incongruent, *t*_(20)_ = −0.541, *p* = 0.595; congruent-incongruent, *t*_(20)_ = 1.348, *p* = 0.193). As a next step, we analyzed iCoh in a left and right intrahemispheric network between visual and somatosensory cortices, respectively (Fig. 4*A*, arrows). In the left hemisphere, we observed a decrease in iCoh for congruent, but not for incongruent trials, resulting in a significant difference between congruent and incongruent conditions (Fig. 4*B*, left; Bonferroni-corrected *p* values; congruent, *t*_(20)_ = −4.287, *p* = 0.001; incongruent, *t*_(20)_ = −0.130, *p* = 2.694; congruent-incongruent, *t*_(20)_ = −2.882, *p* = 0.027). In the right hemisphere, iCoh did not significantly change for either congruent or incongruent trials (Fig. 4*B*, right; Bonferroni-corrected *p* values; congruent, *t*_(20)_ = 0.621, *p* = 1.624; incongruent, *t*_(20)_ = 0.363, *p* = 2.162; congruent-incongruent, *t*_(20)_ = 0.177, *p* = 2.585).

10.1523/ENEURO.0101-19.2019.f4-1 Figure 4-1 Average power change of 40 Hz activity in visual and somatosensory cortices. Average power change in left visual (blue) and somatosensory cortices (red) for the left and right hemispheres. Paired-sample *t* tests showed that none of these changes represent a significant deviation from zero (all uncorrected *p* > 0.15). Although, descriptively, 40 Hz power increased in right hemisphere and decreased in left hemisphere, comparisons between left and right visual and somatosensory cortices, respectively, were nonsignificant (all uncorrected *p* > 0.1). Download Figure 4-1, EPS file.

10.1523/ENEURO.0101-19.2019.f4-2 Figure 4-2 Correlation between power in and imaginary coherence between visual and somatosensory cortices at 40 Hz. Scatter plots depict differences incongruent − incongruent in *z*-scored and normalized power/iCoh. Top left, No significant correlation between power in left visual cortex and imaginary coherence between left visual and somatosensory cortices. Top right, No significant correlation between power in right visual cortex and imaginary coherence between right visual and somatosensory cortices. Bottom left, No significant correlation between power in left somatosensory cortex and imaginary coherence between left visual and somatosensory cortices. Top right, Significant correlation between power in right somatosensory cortex and imaginary coherence between right visual and somatosensory cortices. Download Figure 4-2, EPS file.

## Discussion

We investigated the role of long-range gamma synchronization between sensory cortices in multisensory perception. In a cross-modal matching task, participants compared amplitude information across visual and somatosensory modalities, which we assumed to rely on direct interaction between sensory cortices. In an attempt to modulate such direct cross talk between the senses, we used multi-electrode tACS to influence coupling between sensory gamma oscillations in a phase-specific as well as a frequency-specific manner. We show that gamma tACS, but not alpha tACS, over sensory cortices targeted by sensory input modulated the degree of multisensory congruence enhancement. Stimulation over the same areas in the hemisphere not targeted by sensory stimulation did not have a significant influence on congruence enhancement, but showed opposite effects for alpha and gamma stimulation. Finally, we reanalyzed EEG data from a comparable task and found imaginary coherence between visual and somatosensory cortices to be modulated by cross-modal congruence.

### Cross-modal matching involves communication, not feature binding, between modalities

In our paradigm, cross-modal matching between congruent stimuli was associated with speeded responses when compared with the matching of incongruent stimuli. This behavioral benefit of cross-modal congruence is well in line with previous studies that consistently showed faster responses and elevated accuracy of detecting or discriminating congruent multisensory stimuli (Bolognini et al., 2005; Schneider et al., 2008b; Göschl et al., 2014; Misselhorn et al., 2016). It was proposed that these behavioral benefits might arise because of enhanced cross-modal binding mediated by elevated synchrony between sensory gamma oscillations (Senkowski et al., 2008; Keil and Senkowski, 2018). Accordingly, congruent multisensory stimuli should induce stronger cross-modal coupling when compared with incongruent stimuli, and synchronizing sensory cortices by tACS should be beneficial for the processing of congruent, but not incongruent, multisensory inputs. In our data, however, we found the opposite pattern of results. Compared with sham stimulation, in-phase gamma stimulation over cortices targeted by sensory input led to a significant reduction of the congruence effect. Specifically, responses in congruent trials were slowed down, whereas responses in incongruent trials were as fast as under sham stimulation. While anti-phase gamma stimulation showed a similar pattern, this effect was not significant on the level of the ANOVA. In a follow-up analysis using nonparametric permutation statistics to compare CDFs of RT data, we found both in-phase and anti-phase gamma stimulation over task-relevant sensory cortices to delay responses to congruent, but not incongruent, stimuli. Interestingly, in-phase stimulation showed an earlier peak of difference with sham stimulation than with anti-phase stimulation, leading to a significantly stronger delay of responses for in-phase compared with anti-phase tACS for responses between ∼400 and ∼700 ms.

Additionally, we analyzed cortical 40 Hz activity in EEG data that we had previously recorded during a similar task (Misselhorn et al., 2019). In a source space analysis, we found condition-averaged power to be relatively suppressed in the left hemisphere and relatively increased in the right hemisphere. Although these global trends were also reflected in visual and somatosensory cortices, statistical comparison did not show significant changes in power in either left or right hemisphere. Furthermore, power was not correlated with coupling in the left visual and somatosensory cortices as well as in right visual cortex. Only power in right somatosensory cortex showed a negative correlation with imaginary coherence. Importantly, we did not find significant modulations of power by cross-modal congruence. In the analysis of coupling in the right hemisphere, we could not detect any effect of cross-modal congruence. This absence of modulation in 40 Hz iCoh, however, might be confounded by the negative correlation between iCoh and power and is not interpreted (Bastos and Schoffelen, 2016). Importantly, we could show that 40 Hz iCoh between visual and somatosensory cortices of the left hemisphere was significantly decreased during cross-modal matching for congruent stimuli, but not for incongruent stimuli.

This pattern of results cannot be explained with the binding-by-synchrony hypothesis (Engel et al., 2001; Senkowski et al., 2008). In this view, synchrony serves as a tag that binds neuronal assemblies that code for the same, potentially multisensory, object or feature. Behavioral benefits would thus arise because of a strengthened or more stable representation of the multisensory object that facilitates further processing. This rather passive or static perspective on cross-modal interactions does not seem to apply to the paradigm used here, where active matching between the senses was required. We thus propose to interpret our results in the context of the communication through coherence hypothesis, which represents an extension of the binding-by-synchrony hypothesis by highlighting the dynamic routing capabilities of synchronizing distant cell assemblies (Fries, 2009, 2015). In this perspective, coherence between gamma oscillations enables communication between interconnected cortical areas that allows for information transfer. We speculate that perceiving incongruent stimuli was associated with an increased need for communication between sensory areas reflected by stable levels of cross-modal gamma coherence over time, which were seen in the reanalysis of EEG data. Congruent stimuli, on the other hand, were related to a fast decoupling between sensory cortices, which would correspond to a termination of direct communication, perhaps to the effect of gating information flow to higher-order cortical areas such as the parietal lobes. Synchronizing sensory cortices by 40 Hz tACS is proposed to have hampered such sensory decoupling and thereby reduced the amount of behavioral benefit for congruent stimuli. Collectively, these findings lend support to the idea that gamma oscillations play a critical role in structuring interactions and likely communication among sensory cortical areas.

### Limited phase specificity of behavioral tACS effects

As described above, we found in-phase gamma stimulation to exhibit significant effects on processing cross-modally congruent stimuli in an ANOVA. In a follow-up analysis, however, we show that in-phase and anti-phase stimulation differ in a small fraction of the RT distribution quantitatively, but not as hypothesized qualitatively. That is, anti-phase gamma stimulation also reduced the behavioral benefit of cross-modal congruence. This result stands in contrast to earlier findings that describe differential effects of in-phase and anti-phase stimulation on cortical coupling and/or behavior (Polanía et al., 2012; Helfrich et al., 2014; Schwab et al., 2019). One explanation for these phase-unspecific effects might be that tACS boosted the power of ongoing sensory gamma oscillations without deflection of phase. While, in principle, this is possible, the current pattern of results is unlikely to be related to enhanced gamma power. First, the power of sensory gamma oscillations was previously shown to be associated with faster response times for both visual and tactile stimuli (Krebber et al., 2015; van Es and Schoffelen, 2019). Thus, enhanced sensory gamma power should speed, not delay, responses. Second, cross-modally congruent stimuli have been repeatedly shown to induce stronger gamma responses than incongruent stimuli (Senkowski et al., 2005, 2007; Yuval-Greenberg and Deouell, 2007; Schneider et al., 2008a; Krebber et al., 2015). Thus, further boosting of gamma power should have aided not hindered responding to congruent stimuli.

Alternatively, we should question the overarching assumptions that were made in this and many other tACS studies designed to modulate interareal coupling (Saturnino et al., 2017). That is, expecting effects of opposite sign for in-phase and anti-phase stimulation relies on a number of assumptions that might not necessarily hold, most importantly that (1) the targeted process actually exhibits (near) zero-phase properties and that (2) cortical activity can be affected by tACS without phase lag. The targeted process here is direct communication between sensory areas, which has been described, but remains poorly understood (Cappe and Barone, 2005; Kayser et al., 2008). Importantly, long-range interactions between distinct sensory cortices involve conduction delays that might differ with respect to task parameters, rendering (near) zero-lag coupling unlikely (Raij et al., 2010). Furthermore, tACS might not affect cortical activity with the same phase lag over participants (Riecke et al., 2015; Asamoah et al., 2019). Although electric currents reach cortical structures without significant temporal lag, differences in the anatomy of the skull and, most importantly, differences in cortical folding might lead to different preferred phases of tACS (Liu et al., 2018). That is, cortical target orientations might differ by up to 180° across participants, which would render effective in-phase and anti-phase stimulation to be opposite for extreme cases. In our data, we suggest that the lack of phase specificity might have arisen due to suboptimal fit of individual phase lags with the 0° and 180° conditions of stimulation that were used. In effect, both in-phase and anti-phase stimulation might have suited some, but not all, participants’ anatomy and physiology. Testing this speculation, however, requires a larger set of phase lag conditions and, optimally, physiologic data to relate to.

### Contralateral alpha/gamma dynamics regulate cortical excitability

To control for unspecific effects of tACS, we used stimulation over visual and somatosensory areas in the hemisphere not targeted by lateralized sensory stimuli as an active control. Our results showed global modulations of response times that were specific with respect to frequency, but unspecific with respect to the task conditions. Note, however, that these effects were small and were significant only when evaluating RT distributions with permutation statistics. While gamma stimulation showed overall slowing of responses, alpha stimulation showed overall speeding of responses. These findings can be related to the functionally opposing roles of alpha and gamma oscillations in cortex, as follows: while gamma oscillations are enhanced from activated cortical areas (Donner and Siegel, 2011), alpha oscillations predominate in task-irrelevant cortical regions (Jensen and Mazaheri, 2010). This view is supported by a negative or positive correlation of EEG gamma and alpha power, respectively, with the BOLD signal (Mulert et al., 2010; Scheeringa et al., 2011). Importantly, the level of ongoing alpha activity could be shown to be a readout of cortical excitability as determined by transcranial magnetic stimulation (Romei et al., 2008). Cortical excitability, as controlled by alpha/gamma dynamics, is also discussed as a mechanism underlying top–down control of perceptual processes (Jensen and Mazaheri, 2010; Bonnefond and Jensen, 2015). For instance, cued spatial attention led to a lateralization of prestimulus alpha power to the task-irrelevant hemisphere, while stimulus-related gamma activity was lateralized to the task-relevant hemisphere (Marshall et al., 2015). In our tACS experiment, stimuli were presented lateralized and pretrial cues were used to guide the spatial attention of participants. We propose that reducing cortical excitability of the task-irrelevant hemisphere through alpha stimulation improved processing in the task-relevant hemisphere. Conversely, gamma stimulation might have increased the excitability of the task-irrelevant hemisphere and thereby impeded processing in the task-relevant hemisphere. A similar result has recently been obtained for unilateral stimulation over temporoparietal cortex in a dichotic listening task (Wöstmann et al., 2018). In their study, alpha tACS decreased the recall of contralateral items while gamma tACS showed the opposite effect. Our results, thus, add to the abundant literature that suggests an important role of alpha/gamma dynamics in modulating cortical excitability.

### Limitations and future directions

The main limitation, which applies to most current tACS experiments, is the lack of concurrent measurement of the underlying physiology. Due to nonlinear artifacts in the EEG, acute physiologic effects of tACS can currently not be investigated with EEG (Noury et al., 2016; Noury and Siegel, 2017). Yet, investigating aftereffects represents a viable alternative that can be used to observe the lingering effects of stimulation (Schwab et al., 2019). Due to the unavailability of these data, we cannot provide details of the physiologic underpinnings of the behavioral modulation described here. To compensate for the lack of direct physiologic data, we reanalyzed data from a similar task. Although this allows some inference and certainly can guide the interpretation of the behavioral results, its explanatory power is limited because separate groups were investigated. We therefore could not investigate any direct relations between the EEG and tACS-modulated behavior. As outlined above, another limitation was the choice of two phase lags only. Future studies should use more phase lags to identify optimal phase lags that likely differ across individuals.

### Conclusions

While there is ample evidence for the active role of gamma oscillations for interactions within sensory streams, we provide evidence for the importance of coordinated sensory gamma oscillations for direct cross-modal interactions. Both tACS behavior and EEG results converged in showing that corticocortical coupling of sensory gamma oscillations is likely involved in processing cross-modal congruence, a fundamental computation in solving the multisensory binding problem. Interestingly, the pattern of results suggests that these sensory interactions should be interpreted in terms of communication rather than feature binding. We propose that the evaluation of cross-modal congruence involves direct cross-modal communication routed by flexible and dynamic coupling and decoupling of sensory gamma oscillations between sensory cortices. As a working hypothesis, we further suggest that phase-coupled gamma oscillations might more generally provide a functional scaffold for cross-modal communication.

## Synthesis

Reviewing Editor: Satu Palva, University of Helsinki Decisions are customarily a result of the Reviewing Editor and the peer reviewers coming together and discussing their recommendations until a consensus is reached. When revisions are invited, a fact-based synthesis statement explaining their decision and outlining what is needed to prepare a revision will be listed below.

The current manuscript is a resubmission of a previously rejected manuscript. The authors took great care to address previously raised issues and the core of the paper (results, also discussion) are now much easier to follow As such, the paper has gained a lot in clarity, Yet, there are a few lingering points in terms of synopsis of the results (abstract) and data presentation.

Major points

1) Abstract: The addition of detail helps the reader to orient in terms of what has been done, but I still struggle with several points in this section. Why starting the description of the results with the active control (“stimulation of the hemisphere nor targeted by sensory input”), and not with the main condition (“tACS and sensory input targeted at the same hemisphere”)? This is counterintuitive and confusing, even more so as the results and discussion section do not follow this counterintuitive order of presentation. Aligning the abstract more with the logic of the results/ discussion sections would help reading and the understanding of the present contribution (which is not straightforward as not as expected). May be it would also help to emphasize that the results are contrary to expectations, as well as to better articulate why and the ensuing re-interpretations (communication instead of binding) in light of the results. The abstract could/ should convey the main points of this manuscript better to help entry into it.

2) The differentiation between “corticocortical communication” and “binding” should be clarified. Isn't the first the mechanism underlying the second? It becomes clearer in the course of the discussion, but I find the statement too brief for the abstract.

3) Throughout the manuscript, the authors cite “Anonymous, 2019” when referring their previous (unpublished?) EEG work that they re-analyse here to inform the interpretations of the tACS results. For instance, on line 225, I read: “An exhaustive description of experimental procedure and data can be found in (Anonymous, 2019).” However, it is unclear where “Anonymous, 2019” can be found? Please specific how to access this info. If not accessible, it is imperative that the authors lay out in detail all aspects of this work (if not done so already). If done so, please make this clear (the reference to “Anonymous, 2019” does not help in this respect as it implies only partial info is provided here).

5) Methods: processing of EEG analysis: The text only refers to analysis of the congruent condition (lines 281 ad 282). What about incongruent trials? They must have also been included in the analyses? Please specify.

6) Discussion: third section: Its sub-title: “Phase-specificity of behavioral tACS effects” does not to match the conclusions, which seem to emphasize a “limited” phase-specificity? Could the title be adapted accordingly, e.g reworded to “Limited phase-specificity of behavioral tACS effects” to help reading?

7) The naming of the figures and tables was confusing. Please use integers (e.g. Figure 2) for the main figures and something like “Supplementary Figure 1” for the other figures and tables. Similarly, it was not clear which figure is “Figure 2-1”. 4 Also the figure illustrating the results of the tACS-side effect questionnaire (referred to as Fig 2-1 in the text) and Figure 4-1 are missing. These Figures should be provided.

Minor

Figure 2: In the caption, the last sentence refers to panel “G”, not “D”.

Line 391: I guess this not refers to Figure 2-2”, not “Figure 2-1”.

Line 418f: Is this an interpretation of the visual inspection of the plot or did you formally compare 40Hz power between the hemispheres. Similarly, in line 480 you refer to this finding, but I could not find a justification for this claim.

## Author Response

Reply to the reviewers

We thank the reviewers for their comments and are pleased to note that all issues relating to the structure of the manuscript and our line of reasoning in the discussion could be resolved. This review helped us to fine-tune the abstract, elaborate conceptually with respect to the differentiation between “binding” and “communication” and eliminate some remaining errors/typos. All edits are marked in yellow. We apologize for the confusion with respect to the figures and extended data. In the revised manuscript, we included an additional presentation of the figures to ensure that all important information is accessible for the reviewers.

Major

“Abstract: The addition of detail helps the reader to orient in terms of what has been done, but I still struggle with several points in this section. Why starting the description of the results with the active control (“stimulation of the hemisphere nor targeted by sensory input”), and not with the main condition (“tACS and sensory input targeted at the same hemisphere”)? This is counterintuitive and confusing, even more so as the results and discussion section do not follow this counterintuitive order of presentation. Aligning the abstract more with the logic of the results/ discussion sections would help reading and the understanding of the present contribution (which is not straightforward as not as expected). Maybe it would also help to emphasize that the results are contrary to expectations, as well as to better articulate why and the ensuing re-interpretations (communication instead of binding) in light of the results. The abstract could/ should convey the main points of this manuscript better to help entry into it.”

Reply: We agree that the unexpected nature of our results is key to understanding the change of perspective taken in the discussion and conclusion. In the revised abstract, we tried to incorporate this information without reducing the amount of detail on the methods, which are no less important to grasp the scope of our study (ll. 9-23). As suggested, we adapted the order of result presentation in order to fit the results and discussion sections of the manuscript.

“The differentiation between “corticocortical communication” and “binding” should be clarified. Isn't the first the mechanism underlying the second? It becomes clearer in the course of the discussion, but I find the statement too brief for the abstract.”

Reply: We thank the reviewer for pointing out this inaccuracy in wording and explanation - this differentiation is central to our discussion and thus deserves full attention. When referring to “binding”, we really mean “feature binding” which entails a rather static, representational perspective on cortical processes. In demarcation to this view, “communication” is understood as a consequence of a dynamical routing process instantiated by neuronal coherence. Importantly, “feature binding” is taken to signify neuronal tagging while “communication” is taken to implicate information transfer. Thereby, although having the same mechanistic ground, these two perspectives greatly differ with respect to the underlying purpose of the process. We changed wording from “binding” to “feature binding” where it was missing (l.20, l.470, l. 628) and added a more detailed demarcation of the two concepts in the discussion (ll. 511-520). In the abstract, however, we lack the space to elaborate on this conclusion. In the revised version of the abstract, we think that this statement is now clearer because we highlighted that the results

were unexpected and thus cannot support the concept that gave rise to the hypothesized pattern of results.

“Throughout the manuscript, the authors cite “Anonymous, 2019” when referring their previous (unpublished?) EEG work that they re-analyse here to inform the interpretations of the tACS results. For instance, on line 225, I read: “An exhaustive description of experimental procedure and data can be found in (Anonymous, 2019).” However, it is unclear where “Anonymous, 2019” can be found? Please specific how to access this info. If not accessible, it is imperative that the authors lay out in detail all aspects of this work (if not done so already). If done so, please make this clear (the reference to “Anonymous, 2019” does not help in this respect as it implies only partial info is provided here).”

Reply: Due to the double-blind type of review, we anonymized our identity using this label. The referenced work is easily accessible as it is published under open access in a peer-reviewed journal. We are confident that sufficient information on how EEG data was recorded and processed is given in this manuscript. However, if the reviewers think that evaluating this resource is important for their decision, we would be willing to reveal our identity and thus grant access to this paper.

“Methods: processing of EEG analysis: The text only refers to analysis of the congruent condition (lines 281 ad 282). What about incongruent trials? They must have also been included in the analyses? Please specify.”

Reply: This point was not explicitly stated, but of course we analyzed and compared both congruent and incongruent trials. For completeness, we added a sentence to clarify this matter (see ll. 285).

“Discussion: third section: Its sub-title: “Phase-specificity of behavioral tACS effects” does not to match the conclusions, which seem to emphasize a “limited” phase-specificity? Could the title be adapted accordingly, e.g reworded to “Limited phase-specificity of behavioral tACS effects” to help reading?”

Reply: Thank you for the suggestion, we changed the wording accordingly (l. 534).

“The naming of the figures and tables was confusing. Please use integers (e.g. Figure 2) for the main figures and something like “Supplementary Figure 1” for the other figures and tables. Similarly, it was not clear which figure is “Figure 2-1”. 4 Also the figure illustrating the results of the tACS-side effect questionnaire (referred to as Fig 2-1 in the text) and Figure 4-1 are missing. These Figures should be provided.”

Reply: The naming of figures follows the instructions given on eNeuro's webpage (https://www.eneuro.org/content/preparing-manuscript), which reads:

“eNeuro does not accept supplemental material or appendices.”

and

“Only Extended Data directly related to, or supporting figures or tables and corresponding figure legends are allowed. Extended Data should be labeled as Figure 1-1, Figure 1-2, Table 1-1, Table 1-2, etc., so they indicate which figure or table they are supporting (i.e. Extended Data table supporting Figure 5 labeled as Figure 5-1).”

In the legends section, however, we falsely named Figure 2-1 as Figure 2-2, which does not exist. Thus, the title of extended data for Figure 2 should read ‘Figure 2-1. Side effects of tACS.’ (see l. 792).

We are sorry to hear that Figure 2-1 and Figure 4-1 were missing in the manuscript, although they were uploaded as instructed. In order to prevent this from happening again, we added all figures to the Word document after the legends section.

Minor

“Figure 2: In the caption, the last sentence refers to panel “G”, not “D”.”

Reply: Thank you for noting (l. 788)!

“Line 391: I guess this not refers to Figure 2-2”, not “Figure 2-1”.”

Reply: The in-text reference to Figure 2-1 is correct, thus, the title of the figure legends should read ‘Figure 2-1. Side effects of tACS.’ (see l. 792).

“Line 418f: Is this an interpretation of the visual inspection of the plot or did you formally compare 40Hz power between the hemispheres. Similarly, in line 480 you refer to this finding, but I could not find a justification for this claim.”

Reply: We added a statistical analysis of hemispheric differences in 40 Hz power change to the revised manuscript as extended data (Figure 4-1; ll. 828-833 and l. 859). Descriptively, hemispheres show opposed directions of power change (left decrease, right increase). Statistical comparisons, however, were non-significant. In the results and discussion sections, we now present the results from all control analyses with greater detail (ll. 423-439 and ll. 495-509).
