# Supplementary material for: Synchronization of Sensory Gamma Oscillations Promotes Multisensory Communication
Source: eNeuro. 2019 Oct 23;6(5):ENEURO.0101-19.2019. doi: 10.1523/ENEURO.0101-19.2019 (PMC6873160; doi:10.1523/ENEURO.0101-19.2019)
Supplement: Table 1-1 — : Complete and reduced ANOVA results from behavior in tACS experiment Download Table 1-1, DOC file. [file sup_enu-eN-NWR-0101-19-s02.doc]

**Table 1**-1. Complete and reduced ANOVA results from behavior in tACS experiment.

| **Complete ANOVA on accuracy.** | | |  |
| --- | --- | --- | --- |
| factor | F | p | 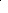 |
| HEMISPHERE | 0.741 | 0.400 | 0.038 |
| FREQUENCY | 0.652 | 0.429 | 0.033 |
| STIMULATION | 0.285 | 0.752 | 0.015 |
| CONGRUENCE | 10.122 | 0.005 | 0.348 |
| HEMISPHERE x FREQUENCY | 1.383 | 0.254 | 0.068 |
| HEMISPHERE x STIMULATION | 1.589 | 0.223 | 0.077 |
| FREQUENCY x STIMULATION | 1.821 | 0.176 | 0.087 |
| HEMISPHERE x FREQUENCY x STIMULATION | 2.783 | 0.074 | 0.128 |
| HEMISPHERE x CONGRUENCE | 1.833 | 0.192 | 0.088 |
| FREQUENCY x CONGRUENCE | 2.621 | 0.122 | 0.121 |
| HEMISPHERE x FREQUENCY x CONGRUENCE | 0.130 | 0.723 | 0.007 |
| STIMULATION x CONGRUENCE | 0.596 | 0.556 | 0.030 |
| HEMISPHERE x STIMULATION x CONGRUENCE | 1.494 | 0.239 | 0.073 |
| FREQUENCY x STIMULATION x CONGRUENCE | 0.635 | 0.532 | 0.032 |
| HEMISPHERE x FREQUENCY x STIMULATION x CONGRUENCE | 0.587 | 0.559 | 0.030 |

| **Complete ANOVA on response times.** | | |  |
| --- | --- | --- | --- |
| factor | F | p | 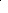 |
| HEMISPHERE | 0.355 | 0.558 | 0.018 |
| FREQUENCY | 0.02 | 0.888 | 0.001 |
| STIMULATION | 0.004 | 0.994 | 0.000 |
| CONGRUENCE | 34.659 | 0.000 | 0.646 |
| HEMISPHERE x FREQUENCY | 0.074 | 0.789 | 0.004 |
| HEMISPHERE x STIMULATION | 0.057 | 0.934 | 0.003 |
| FREQUENCY x STIMULATION | 2.626 | 0.092 | 0.121 |
| HEMISPHERE x FREQUENCY x STIMULATION | 1.543 | 0.229 | 0.075 |
| HEMISPHERE x CONGRUENCE | 0.140 | 0.713 | 0.007 |
| FREQUENCY x CONGRUENCE | 0.013 | 0.910 | 0.001 |
| HEMISPHERE x FREQUENCY x CONGRUENCE | 0.004 | 0.949 | 0.000 |
| STIMULATION x CONGRUENCE | 4.199 | 0.032 | 0.181 |
| HEMISPHERE x STIMULATION x CONGRUENCE | 0.256 | 0.728 | 0.013 |
| FREQUENCY x STIMULATION x CONGRUENCE | 4.089 | 0.027 | 0.177 |
| HEMISPHERE x FREQUENCY x STIMULATION x CONGRUENCE | 4.862 | 0.015 | 0.204 |

| **Reduced ANOVA on response times: Different HEMISPHEREs.** | |  |  |
| --- | --- | --- | --- |
| factor | F | p | 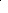 |
| FREQUENCY | 0.030 | 0.864 | 0.002 |
| STIMULATION | 0.010 | 0.988 | 0.001 |
| CONGRUENCE | 24.803 | 0.000 | 0.566 |
| FREQUENCY x STIMULATION | 3.771 | 0.038 | 0.166 |
| FREQUENCY x CONGRUENCE | 0.015 | 0.904 | 0.001 |
| STIMULATION x CONGRUENCE | 3.404 | 0.054 | 0.152 |
| FREQUENCY x STIMULATION x CONGRUENCE | 0.163 | 0.841 | 0.008 |

| **Reduced ANOVA on response times: Same HEMISPHEREs.** | | |  |
| --- | --- | --- | --- |
| factor | F | p | 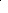 |
| FREQUENCY | 0.012 | 0.913 | 0.001 |
| STIMULATION | 0.006 | 0.992 | 0.000 |
| CONGRUENCE | 40.028 | 0.000 | 0.678 |
| FREQUENCY x STIMULATION | 1.459 | 0.246 | 0.071 |
| FREQUENCY x CONGRUENCE | 0.001 | 0.971 | 0.000 |
| STIMULATION x CONGRUENCE | 1.239 | 0.300 | 0.061 |
| FREQUENCY x STIMULATION x CONGRUENCE | 7.548 | 0.002 | 0.284 |

| **Reduced ANOVA on response times: Same HEMISPHERE, alpha FREQUENCY.** | | |  |  |
| --- | --- | --- | --- | --- |
| factor | F | p | | 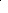 |
| STIMULATION | 0.507 | 0.598 | | 0.026 |
| CONGRUENCE | 43.454 | 0.000 | | 0.696 |
| STIMULATION x CONGRUENCE | 2.079 | 0.145 | | 0.099 |

| **Reduced ANOVA on response times: Same HEMISPHERE, gamma FREQUENCY.** | | |  |  |
| --- | --- | --- | --- | --- |
| factor | F | p | | 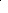 |
| STIMULATION | 0.923 | 0.404 | | 0.046 |
| CONGRUENCE | 26.133 | 0.000 | | 0.579 |
| STIMULATION x CONGRUENCE | 4.578 | 0.018 | | 0.194 |
